# Epidemiology of Self-poisoning with Drug in the Central Anatolian Region in Turkey

**DOI:** 10.7759/cureus.6962

**Published:** 2020-02-12

**Authors:** Onur Karaca, Ayşegül Ertaşkın

**Affiliations:** 1 Anesthesiology and Reanimation, Aksaray University, Aksaray, TUR; 2 Anesthesiology and Reanimation, Aksaray University Training and Research Hospital, Aksaray, TUR

**Keywords:** self poisoning, critical care, epidemiology

## Abstract

Aim: Deliberate self-poisoning (DSP) is a common cause of intensive care hospitalization among young adults and a serious health problem worldwide. Demographic data vary according to geographical and sociocultural characteristics of the regions. In recent years, studies investigating epidemiological features and prognosis of these patients have increased. In our study, we retrospectively examined patients who committed suicide with drugs and were treated in the ICU of our hospital.

Materials and Methods: The files of 148 patients who took drugs or substances for committing suicide and who were hospitalized in the ICU of Aksaray Training and Research Hospital between 2015 and 2019 were examined. Demographic data of the patients, type of the agent used in the suicide, time to reach hospital, treatment methods applied, length of hospital stay, vital signs, complications, need for intubation, and mortality rates were recorded.

Results: Mean age of the 148 patients who took drugs for suicide was 26.7. Female rate was 73%. The most frequently used drug for suicide was paracetamol (34.4%). Antidepressants took the second place and were followed by drugs in the NSAID group. The duration of admission in the hospital after taking the medicine ranged from 1 to 6 h, while it was less than 3 h in 68.2% of the patients. In most suicide patients, the treatment method was in the form of intravenous fluid and supportive therapy (95%). N-acetyl cysteine (paracetamol intoxication) was used in 7% of the patients, an intubation requirement developed in 2.7%, and three patients taking organophosphate died.

Conclusion: In studies conducted in developing countries such as Turkey, female sex (63%-71%) and 25 years of age have been found to be the proportion of the patients (56%-63%), whereas our study found even higher ratios compared to those (73%-66%). In studies conducted in developed countries, most commonly used agents for suicide were benzodiazepines and tricyclic antidepressants, while the most common suicide agent was paracetamol in our study. We believe that the reason for this could be the possibility of accessing the agent without a prescription.

## Introduction

Deliberate self-poisoning (DSP) is a common cause of hospitalization among young adults and a serious health problem worldwide [1-2]. Epidemiological characteristics may vary depending on time and geographical location [3]. DSP accounts 1%-5% of hospital admissions in developed countries and is the most encountered method of suicide [4-5]. After first assessment in the emergency service, 4%-30% of these patients are referred to the ICU in the presence of life-threatening conditions [6-7]. Management of poisoned patients in the ICU requires fast diagnosis and supportive treatment [2, 8-9]. In recent years, epidemiological studies on patients admitted to the ICUs for overdose by self-poisoning in developed countries have been published [4, 10, 11]. To the best of our knowledge, there is no epidemiological study on patients with low sociocultural status and living in the Central Anatolian region. We performed a retrospective observational study of self-poisoned patients admitted to the ICU of Aksaray University Training and Research Hospital (ATRH), which is a university hospital in the Central Anatolian region of Turkey. Our aim was to investigate the age, sex, type of poisoning, exposure to toxic agents, consciousness status at the time of admission, ICU stay, treatment, and prognosis of the patients hospitalized with a diagnosis of DSP.

## Materials and methods

All procedures carried out in studies including human participants were conducted in compliance with the ethical standards of institutional and/or national research committee, the 1964 Helsinki Declaration and its amendments or comparable ethical standards. Ethical approval for the study was obtained from the institutional review board of Aksaray University. Study population consisted of 148 patients admitted for DSP to ATRH ICU, which serves a population of around 450,000, between the 1st of January 2015 and the 31st of December 2019. DSP patients with a life-threatening situation are admitted to the ICU, which is a third stage ICU in the city of Aksaray. Age, sex, duration of ICU, type of self-poisoning (drugs, rat poisons, and pesticides), the way of poisoning (suicides, accidents), treatment, and prognosis of the 148 DSP patients were screened retrospectively and recorded. Statistical analyses were performed using SPSS 22.0 software (SPSS Inc., Chicago, IL). Data analysis was carried out using descriptive and inferential statistical methods: frequency, percentage, mean, and standard deviation (SD). A p-value of less than 0.05 was considered statistically significant.

## Results

**Figure 1:**
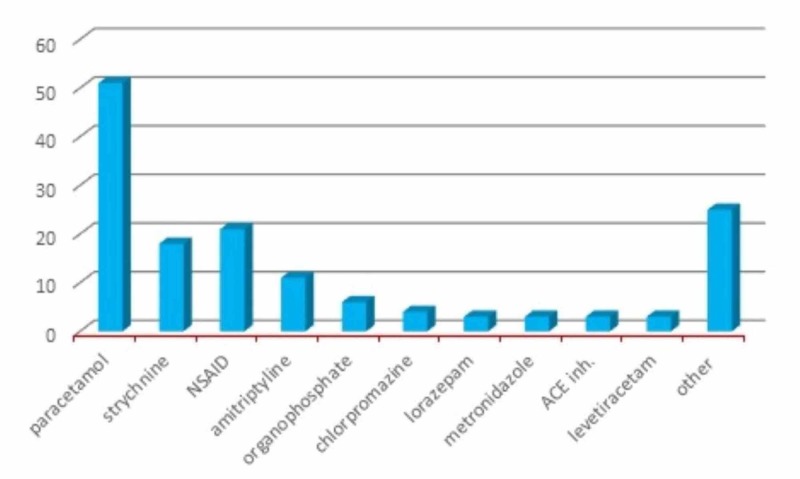
Agents of poisoning.

A total of 148 patients were admitted to the ICU due to DSP. Mean age of the 148 DSP patients was 26.7 (std. dev: 14, range: 17-94). One hundred and eight (73%) patients were female and 98 (66.2%) patients were under 25 years of age (see Table 1). The most common cause of DSP was drugs (83.7%), and paracetamol was the most frequently used agent (34.45%). Agent distribution is summarized in Table 2 and Figure 1. Time interval from DSP to the beginning of treatment ranged between 1 and 6 h, and 68.2% of the patients were admitted in less than 3 h. The treatment delivered in the ICU was mainly symptomatic and supportive, based on vital signs, stabilization, oxygen, and IV liquids. During follow up in the ICU, four patients (2.7%) were intubated due to the development of respiratory insufficiency and needed mechanical ventilation. The only specific antidote used in the ICU was N-acetylcysteine due to poisoning with paracetamol in toxic doses. Three patients who were followed up due to organophosphate poisoning died after having developed multiorgan failure. Findings are summarized in Table 1.

**Table 1:** Demographics and file data of the patients.

Variables (n=148)	n (%)
Sex	
Males (%)	40 (27.1)
Females (%)	108 (72.9)
Age, years (mean)	26.7 (SD 14.1)
Males (mean)	32.2 (SD 23.4)
Females (mean)	24.5 (SD 6.7)
Less than 21 years	73 (49.4)
21-25 years	26 (17.5)
25-35 years	33 (22.3)
More than 35 years	16 (10.8)
Time of admission	
Day time (9 am-9 pm)	33 (22.3)
Night time (9 pm-9 am)	115 (77.7)
Time to presentation after consumption	
Less than 2 hours	51 (34.4)
2-5 hours	79 (53.4)
More than 5 hours	28 (12.2)
Duration of ICU	
Less than 2 days	6 (4)
2-4 days	91 (61.5)
More than 4 days	51 (34.5)
GKS at the time of admission	
13-15	107 (72.3)
<13	41 (27.7)
Mean APACHE II score	11.2 ± 4.7 (range, 6-32)

**Table 2:** Agents of poisoning. *Other agents: lansoprozole, alporolozame, metformin, colchicine, carbamazepine, hydroxyne HCL, duloxetine, keitapine fumarate, ciprofloxacin, bupcopion, fexofenadine, ferro glycol sulfate, ursodeoxic acid, ramipril, propiverine

Agents of poisoning	n (%)
Paracetamol	51 (34.45)
Strychnine	18 (12.16)
NSAID	21 (14.18)
Amitriptyline	11 (7.43)
Organophosphate	6 (4.05)
Chlorpromazine	4 (2.75)
Lorazepam	3 (2.02)
Metronidazole	3 (2.02)
ACE inh.	3 (2.02)
Levetiracetam	3 (2.02)
Others*	25 (16.9)

## Discussion

Though self-poisoning is a common cause of hospital admissions, only a small percent of these patients needs intensive care [1-2]. Despite this fact, high mortality and morbidity risks make early diagnosis and treatment very important. Therefore, etiological and demographical characteristics must be well known. Female rate ranges between 63% and 71% [12-13] and the rate of young patients aged under 25 ranges between 56% and 63% [13-14] according to demographical data in Turkey. These rates are 52%-60% and 25%-29% respectively in developed countries [15-16]. In our study, the female rate was 73% and the rate of young patients was 66.2%. These high rates indicate more sociocultural pressure on women in this region of Turkey and we are of the opinion that the reason for this may be that the rate of early marriage and social pressure on women is high and the sociocultural level is low in the city where we conducted the study.

While general mortality of acute poisonings is low, acute insecticide poisonings and aluminum phosphide poisonings have high mortality rates [17]. In our study, four patients (2.7%) needed mechanical ventilation due to respiratory insufficiency and these results were compatible with previous large trials [18-19]. In addition, three patients diagnosed with organophosphate poisoning died after the development of multiple organ failure. This result is compatible with those found in other studies conducted in Turkey on mortality rates (0.1%-2.9%) [18-20].

Studies conducted in developed countries have shown that benzodiazepine and tricyclic antidepressant use constitutes most of the ICU hospitalizations of patients admitted with a history of DSP [21-22]. In our study, paracetamol use (34.45%) was the most commonly encountered factor for hospitalizations in patients with low sociocultural and socioeconomic characteristics. We think that this situation is due to the easy availability of paracetamol derivatives, and hence, prescription necessity of paracetamol derivatives in this kind of societies may prevent the abuse of these drugs. However, the results of our study are compatible with the results of some studies conducted in developed countries. Studies done in England and Ireland have shown paracetamol use as the most common method of DSP [23-25]. While it emphasizes the variability of drugs used in DSP in different countries, intercountry and interregional tendencies should be reviewed regularly.

Rational and cost-effective treatment strategy is important in the management of poisoned patients physically recovered with only supportive treatment modalities. Like in our study, it is mandatory to know the treatment algorithm of patients with paracetamol intoxications as these intoxications have few antidotes like N-acetylcysteine since N-acetylcysteine use is the most effective treatment method after symptoms and signs appear and before hepatotoxicity occurs [26].

## Conclusions

We described patients with DSP in the Central Anatolian region. Our findings differ from those of the developed countries in terms of younger age and high female rates. Young women are at more risk. Moreover, we found that instead of antidepressants and antipsychotics, paracetamol was the most common agent used in DSP. High treatment cost of DSP due to ICU hospitalization is an important reason for economic burden. It is important to take precautions regarding the easy availability of paracetamol derivatives. In addition, educational effort on people at high risk is the corner stone of management.
